# Proceedings of Odontological Society of Great Britain

**Published:** 1885-01

**Authors:** 


ARTICLE II.

### ODONTOLOGICAL SOCIETY OF GREAT BRITAIN.

At the monthly meeting of this society, held on December 1st, Mr. J. S. Turner, president, in the chair.

The Curator announced that the Council sanctioned the purchase of a very good skull and almost the entire skeleton of a gorilla.

Mr. McAdam, of Hereford, described a mode of regulating teeth by fitting a gold band, and fixing this to the tooth by means of an osteo cement. The band could be furnished with hooks, lugs, or studs, fixed in any required position, and could thus be readily acted upon without the risk of ligatures slipping up under the gum.

Mr. Walter Coffin showed a photograph and models of the mouth of the last surviving member of the Russian Kostroma family, a boy now aged fourteen. He was probably the most hairy member now living of the human race, his face being strongly suggestive of a Scotch terrier. He was almost edentulous, having only two canines in the upper jaw and three incisors in the lower, all temporary teeth.

Dr. St. George Elliot exhibited and presented to the museum some very curious dentures of native Japanese manufacture. They were carved out of hard wood, the the front teeth being made of quartz pebbles carefully ground down, whilst the place of the molars was occupied by copper nails, by means of which most of the mastication was accomplished. Dr. Elliott said the art of making suction plates had been known to the Japanese for two hundred years, though the Chinese had no idea of it.

A communication from Mr. Oakley Coles on the use of Cocaine in Dental Surgery was read by Mr. S. J. Hutchison. He (Mr. Coles) had received a small quantity of this drug, and had made some experiments with it which fully confirmed the statements which had been made with reference to its remarkable anæsthetic properties. One application of a 20 per cent. solution would allay sensitiveness in the dentine, and two applications at intervals of five minutes would suspend for a time the sensitiveness of an exposed pulp. It had been stated that, if used in sufficient quantity and of adequate strength, cocaine would enable an operator to remove a tooth without pain; of this he could not speak from personal knowledge, but there could be no doubt as to its utility in dental surgery. The form in which it could be best applied could only be determined by experience. It was soluble in water, glycerine, chloroform and the essential oils, but not in ether; that which he had been using dissolved in oil of cloves. Possibly it might act best in the cavity of a tooth if used in powder or in the solid crystaline form. This, as well as the limits of its power as a local tnæsthetic, coul only be ascertained by careful experiments.

Mr. S. J. Hutchinson remarked that he found the application of a 20 per cent. solution removes the sensitiveness of an exposed pulp.

Mr. Woodruff and Mr. Hern said they had tried the four per cent. solution used in opthalmic practice, but without any beneficial effect.

Mr. Storer Behnett said he had found a four per cent. solution quite useless. He then tried a mixture of vaseline with 10 per cent. of the alkaloid, and found that even after being sealed up in the cavity for 48 hours, it had produced very little effect on the sensibility. He then tried the same 10 per cent, preparation and left it a week. This had a much better effect, the patient remarking that "the tooth felt numb." He intended to try a 20 per cent. solution of the muriate, which he believed had been found much more efficacious than the uncombined cocaine. At present the price was an obstacle to its being used at all extensively, that of cocaine being 10d., and of the muriate 2s, 6d. a grain.

Mr. J. Bland Sutton, F. R. C. S., then read a paper on "Comparative Dental Pathology."

After giving some details respecting certain cases of dental disease in extinct mammals, Mr. Sutton showed two very interesting specimens of odontomes in marmots. In the first case, four odontomes were found, one in connection with each of the incisor teeth; in the second, a a large odontome was found in connection with one of the lower incisors.

In his paper read twelve months ago, he had called attention to the fact that, comparatively trivial as were the effects of alveolar abscess in man, in animals it not unfrequently proved fatal, through the purulent secretion being inspired, and setting up septic phneumonia. He now related the case of a Kangaroo, the death of which had resulted from general pyæmia secondary to alveolar abscess. The most serious local effects resulting from this cause which he had met with were seen in an Exquimaux dog. An alveolar abscess had arisen in connection with the left lower second molar; necrosis of a considerable portion of the outer surface of the jawbone had followed, so as to lay bare the sockets of the premolar teeth. The burrowing pus had also involved the sectoral tooth, which was necrosed and its fangs exposed. On the right side the sectorial and premolar teeth had disappeared, and their alveoli had undergone absorption.

He had also on the former occasion drawn attention to the fact that premature shedding of teeth in animals might result from constitutional disease. He now gave further examples in support of this. In a crab eating opossum affected with a disease resembling mollities ossium, the teeth were so loose that on detaching the mucous membrane they all came away with it, looking exactly as though they were rows of nails driven for half their length through a strip of leather. Animals affected with rickets were liable to loose their teeth owing to extensive absorption of the alveoli. He showed as example of this the skulls of a small species of lemur and of a marmoset. Ophidian snakes also, when kept in captivity, were very liable to lose their teeth from absorption of the "bone of attachment," by which, in these creatures, the base of the tooth is cemented to the jawbone proper. Besides being secondary to general constitutional disease, premature loss of teeth might also occur as the result of a local inflammatory affection characterised by extensive deposits of tartar, and absorption of the alveoli, with evidence of periostitis.

Cleft palate was a deformity which was not confined to man, but might occur in animals also under certain circumstances. Thus some years ago, several of the lion cubs born in the Zoological Gardens had cleft palates. The parent animals were at that time fed entirely on horse flesh, the bones of whlch were too hard even for the jaws of a lion or tiger; but after goat's flesh had been occasionally substituted, well formed cubs were born, the animals being now able to obtain a proper quantity of earthy phosphate. Frequently recurring parturitions, by causing an increased demand for lime salts, would also tend to produce this deformity in animals, and he was of opinion that the same cause might operate to produce it in man.

The subject of cleft palate in man and animals had been made the theme of some very interesting observations by Professor Paul Albrecht, of Brussels, who endeavoured to show, by reference to cases of cleft palate, that the premaxillary bone is developed from two centres, instead of one, as has been generally supposed. He further points out that in some cases where the cleft in the palate is double, so as to isolate the inner portion of the pre-maxillary bones, the median piece may develop two incisor teeth in each half, and yet an incisor tooth may be found lodged in a socket, and separated from the canine by the maxillo-pre-maxillaro suture. From this, Professor Albrecht argues that man naturally inherits three incisors on each side, but that in the course of development the middle one on either side is suppressed. In these cases of cleft palate with an enlarged median portion there is a greater local supply of blood than usual; this extra supply of nourishment enables the usually suppressed second incisor to be fully developed, and show that that which is usually considered the second incisor is really the third. Cases confirmatory of this view had been met with and recorded by other observers, and it seemed probable that the so-called supernumerary incisors which were not very unfrequently met with might really be due to the persistence of one of these usually abortive teeth.

Comparative Dental Pathology was a wide subject and material was abundant. He hoped, therefore, that on some future occasion he might have an opportunity of bringing before the Society some further facts in connection with it.

A Discussion followed, after which the Society adjourned.—*Dental Record.*

---

ARTICAL III.

